# Spatio‐temporal variation in lifelong telomere dynamics in a long‐term ecological study

**DOI:** 10.1111/1365-2656.12741

**Published:** 2017-09-28

**Authors:** Lewis G. Spurgin, Kat Bebbington, Eleanor A. Fairfield, Martijn Hammers, Jan Komdeur, Terry Burke, Hannah L. Dugdale, David S. Richardson

**Affiliations:** ^1^ School of Biological Sciences University of East Anglia Norwich Research Park UK; ^2^ Groningen Institute for Evolutionary Life Sciences University of Groningen Groningen The Netherlands; ^3^ Department of Animal and Plant Sciences University of Sheffield Sheffield UK; ^4^ School of Biology The Faculty of Biological Sciences University of Leeds Leeds UK; ^5^ Nature Seychelles Roche Caiman Mahé Republic of Seychelles

**Keywords:** biomarkers, intra‐ and inter‐individual variation, life history, senescence, Seychelles warbler, telomere

## Abstract

1. Understanding individual‐level variation in response to the environment is fundamental to understanding life‐history evolution and population dynamics. Telomeres, the protective caps at the ends of chromosomes, shorten in response to oxidative stress, and telomere shortening is correlated with reduced survival and life span. Investigating telomere dynamics may help us quantify individual variation in the costs experienced from social and ecological factors, and enhance our understanding of the dynamics of natural populations.
2. Here, we study spatio‐temporal variation in lifelong telomere dynamics in the Seychelles warbler, *Acrocephalus sechellensis*. We combine long‐term life history and ecological data with a large longitudinal dataset of mean telomere lengths, consisting of 1,808 samples from 22 cohorts born between 1993 and 2014. We provide a detailed analysis of how telomere dynamics vary over individual life spans and cohorts, and with spatio‐temporal variation in the social and ecological environment.
3. We found that telomere length decreases with cross‐sectional and longitudinal measures of age, and most rapidly very early in life. However, both cross‐sectional and longitudinal data suggested that against this overall pattern of shortening, bouts of telomere length increase occur in some individuals. Using a large number of repeated measurements we show statistically that these increases are unlikely to be explained solely by qPCR measurement error.
4. Telomere length varied markedly among cohorts. Telomere length was positively associated with temporal variation in island‐wide insect abundance—a key resource for the insectivorous Seychelles warbler—suggesting that the costs associated with living in harsher environments can be studied by investigating telomere dynamics. We also found evidence for sex‐specific relationships between telomeres and tarsus length, potentially reflecting differential costs of growth.
5. Our long‐term data show that in a natural population, telomere dynamics vary in a complex manner over individual life spans, and across space and time. Variance in telomere dynamics among individuals is the product of a wide array of genetic, parental and environmental factors. Explaining this variation more fully will require the integration of comprehensive long‐term ecological and genetic data from multiple populations and species.

## 1 INTRODUCTION

A major aim of ecologists and evolutionary biologists is to understand why individuals vary in their response to different environmental factors. Identifying this variation in individual responses to the environment is central to understanding variation in fitness (Lindström, 1999), and thus for understanding population and community dynamics (Bolnick et al., 2011). Furthermore, knowledge of the relative impact that different environmental factors exert on individuals, and why individuals may differ in mitigating these costs, is important to understanding evolutionary trade‐offs and life‐history strategies (Stearns, 1992). However, fully quantifying individual‐level variation in costs is impossible in wild systems, and thus effective biomarkers that reflect the physiological consequences of individual‐level experiences are required.

Telomeres have been proposed to be a potential biomarker of such costs (Monaghan, 2014). Telomeres are repetitive DNA sequences at the ends of linear chromosomes that protect against DNA damage. Telomeres generally shorten with age (Barrett, Burke, Hammers, Komdeur, & Richardson, 2013; Müezzinler, Zaineddin, & Brenner, 2013), and there is evidence from a range of taxa that telomere shortening is fastest in early life (e.g. Frenck, Blackburn, & Shannon, 1998; Heidinger et al., 2012). In vitro research has shown that telomere shortening can be accelerated by oxidative stress (Von Zglinicki, 2002), which can be elevated due to many environmental factors. There is evidence from humans, and from captive and wild animal populations, that telomere shortening is influenced by the conditions experienced during both early life and adulthood (Monaghan, 2014; Nettle et al., 2015; Price, Kao, Burgers, Carpenter, & Tyrka, 2013; Reichert, Criscuolo, & Zahn, 2015). Importantly, the extent of telomere shortening is linked to senescence and survival. When telomeres become critically short, cells senesce (Campisi, 2003), and the accumulation of these cells has been suggested to result in organismal senescence and death (Wong et al., 2003). The association between telomere length and senescence has inspired a great deal of recent research into telomere evolutionary ecology, and relationships between telomere dynamics and survival or life span have been documented in wild populations of several species (Barrett et al., 2013; Stier, Reichert, Criscuolo, & Bize, 2015). As yet, however, there is little direct evidence that the relationship between telomere dynamics and survival is causal (Simons, 2015).

Although the causal role of telomeres in senescence and survival is not yet clear, there is mounting evidence that telomeres can act as biomarkers of individual condition and ageing in wild populations. Specifically, telomeres may be able to provide a measure of the ecological stress that an individual has experienced—a signature that can otherwise be difficult to detect (e.g. Asghar et al., 2015; Bebbington et al., 2016; Schultner, Moe, Chastel, Bech, & Kitaysky, 2014). There is also evidence that telomere length, measured longitudinally in individuals, can increase as well as decrease (Bateson & Nettle, 2016; Simons, Stulp, & Nakagawa, 2014), which has important ramifications for our understanding of how telomeres reflect costs. However, such increases in telomere length are often attributed to measurement error (Steenstrup, Hjelmborg, Kark, Christensen, & Aviv, 2013; but see Bateson & Nettle, 2016), and as such their ecological significance is unknown.

Although a considerable amount of effort has been put into studying telomere dynamics in natural populations, our understanding of the forces responsible for explaining variation in telomere length is still limited. Understanding how different factors shape telomere length variation is important, as stated earlier, we can use telomeres as a measure of the costs experienced by individuals, we need to know how different developmental, genetic and ecological variables interact to affect telomeres. Telomere length and rates of shortening can vary according to parental characteristics (Heidinger et al., 2016; Njajou et al., 2007), among sexes (Barrett & Richardson, 2011; Watson et al., 2017) and with a whole host of environmental conditions, including altitude (Stier et al., 2016), heat stress (Simide, Angelier, Gaillard, & Stier, 2016) or infection (Asghar et al., 2015). Recent evidence suggests that telomere dynamics are indeed highly variable over individual life spans, and that even the relationship between telomeres and age can vary markedly among cohorts (Fairlie et al., 2016). To understand which factors best explain variation in telomere dynamics, more studies that incorporate telomere variation over entire life spans with comprehensive, long‐term ecological data are required.

The longitudinal study (since 1986) of the Seychelles warbler (*Acrocephalus sechellensis*) population on Cousin Island provides an excellent system for studying telomere dynamics and senescence patterns in the wild (reviewed in Hammers et al., 2015). Due to the isolated nature of the study population (Komdeur, Piersma, Kraaijeveld, Kraaijeveld‐Smit, & Richardson, 2004) and intensive field monitoring, we have comprehensive ecological and survival data spanning many years (see Section 2, below). Environmental conditions and population density on Cousin Island vary across space and time due to weather‐induced changes in foliage cover and insect prey availability (Van de Crommenacker, Komdeur, Burke, & Richardson, 2011). Variation in oxidative stress experienced by individuals is associated with territory quality (Van de Crommenacker et al., 2011). However, the evidence that individual survival and life span is associated with spatial variation in early life territory quality or local density is equivocal and confounded by variation in subsequent life‐history parameters (Brouwer, Richardson, Eikenaar, & Komdeur, 2006; Hammers, Richardson, Burke, & Komdeur, 2013). There is also variation in the social environment that individual Seychelles warblers experience. Facultative cooperative breeding occurs in this species (Komdeur, 1994; Richardson, 2007; Richardson, Komdeur, & Burke, 2003), and the presence of helpers (but not other resident non‐helpers) in the natal territory is associated with increased survival of offspring later in life (Brouwer, Richardson, & Komdeur, 2012).

Importantly, we have an established protocol for assessing telomere length in the Seychelles warbler (Barrett et al., 2012; Bebbington et al., 2016). Furthermore, telomere dynamics predict survival independently of age (Barrett et al., 2013) and telomere length is negatively associated with inbreeding (Bebbington et al., 2016), suggesting that individual variation in telomere length is ecologically relevant in this species. Thus, we have an excellent system in which to determine the impact of different social and environmental conditions experienced by individuals, and to assess how these costs vary over space and time.

In this study, we test how lifelong telomere dynamics are related to environmental variation across 22 Seychelles warbler cohorts. We first study how telomere length and rates of shortening are related to age and sex across all life stages, and how this relationship varies among cohorts, in order to gain an in‐depth understanding of the temporal dynamics of telomere changes. We then examine, within individuals, how telomere length changes with age, and statistically test whether observed increases in telomere length over individual life spans are larger than can be accounted for by measurement error. Finally, we test how telomere length and shortening are related to a wide range of social and environmental variables in order to gain a fuller understanding of the forces driving telomere dynamics in natural populations.

## 2 MATERIALS AND METHODS

### 2.1 Study species and sampling

The Seychelles warbler is a small (*c*. 15 g), insectivorous passerine bird with a mean life expectancy of 5.5 years at fledging (Hammers et al., 2013). The population of *c*. 320 adult birds on Cousin Island (04′20′S, 55′40′E) has been intensively studied since 1986 (Komdeur, 1992; Richardson, Burke, & Komdeur, 2003; Spurgin et al., 2014). This species’ main breeding season runs from June to September (although a small proportion of pairs also breed between January and March), when the breeding females on many of the *c*. 110 territories will attempt to breed, laying one or, rarely, two or three eggs (Komdeur, Bullock, & Rands, 1991). Breeding attempts are often unsuccessful, and as a result of this low reproductive output, and higher mortality in first‐year birds (39% in first‐year birds vs. 16% in adults; Brouwer et al., 2006), cohort sizes in the Seychelles warbler are typically small (<50; Table S1). The 22 hatch year cohorts used in this study cover 1993–2014—the time period during which our data and sampling are most complete.

The majority (96%) of individuals are ringed (with an individually numbered metal ring and unique combination of colour rings) within the first year of life, and so are of known age. We aged all birds using information on eye colour at first capture (Komdeur, 1991) and previous capture history (Richardson, Burke, et al., 2003). Within the first year of life, birds are classified as nestlings less than 1 month old (rounded to 1 month for analyses), fledglings less than 6 months old (rounded to 6 months) or subadults up to 1 year old (rounded to 10 months). Ages for adult birds were rounded to the nearest year. As Seychelles warblers are non‐migratory endemics naturally confined to the island (Komdeur et al., 2004), an extensive biannual census of birds on Cousin during each breeding season gives accurate measures of local density, social status (e.g. breeder, helper, non‐helper) and individual survival (Barrett et al., 2013; Van de Crommenacker, Komdeur, & Richardson, 2011). Full details of monitoring methods can be found in Brouwer et al. (2012).

Seychelles warblers are highly territorial and all territories were mapped during each main breeding season using detailed observational data on foraging and territorial defence behaviour (Richardson, Burke, et al., 2003). Territory quality is calculated based on territory size, foliage cover and insect abundance (Komdeur, 1992). Where territory quality estimates were not available for a specific year we used the average value for that territory across years (Hammers et al., 2013; see Komdeur, Burke, Dugdale, & Richardson, 2016 for an explanation of how territory quality varies on Cousin Island). Cousin is subject to considerable intra‐ and inter‐annual variation in rainfall and, consequently, insect availability (Komdeur et al., 2016). Such island‐wide temporal variation may override the effects of variation in individual territory quality across the island. As an estimate of seasonal variation in food availability, we calculated an index of the abundance of insects across the entire island during each main breeding season (referred to hereafter as “insect abundance”). This index is calculated as the mean number of insects found per unit leaf area over all monthly surveys carried out on the island in a main breeding season.

Each time a bird is caught on Cousin a range of morphometric measurements are taken, including body mass and tarsus length (to the nearest 0.1 g and 0.1 mm respectively). A blood sample (*c*. 25 μl) is taken via brachial venipuncture, and stored at room temperature in 1 ml of absolute ethanol in a 1.5‐ml screw‐cap microfuge tube.

### 2.2 Molecular methods

For each sample, genomic DNA was extracted from a *c*. 2‐mm^2^ flake of preserved blood using the DNeasy Blood and Tissue Kit (Qiagen), following the manufacturer's protocol, with the modification of overnight lysis at 37°C and a final DNA elution volume of 80 μl. Sex was determined using the PCR‐based method outlined by Griffiths, Double, Orr, and Dawson (1998). Prior to telomere analysis, DNA concentration and purity were quantified using a NanoDrop 8000 Spectrophotometer (ThermoScientific). The following thresholds were applied before samples were included for further analysis: (1) DNA concentration must be at least 15 ng/μl (based on a mean of three measurements), (2) the 260/280 absorbance ratio has to be between 1.8 and 2.0 for acceptable DNA purity, and (3) the 260/230 absorbance ratio must be higher than 1.8. DNA integrity was further validated by visualisation with ethidium bromide after electrophoresis on a 1.2% agarose gel, and all samples with evidence of DNA degradation were re‐extracted or excluded. We found no evidence of DNA degradation in older samples (Figure S1). All DNA extractions that passed the above criteria were diluted to 3.3 ng/μl before telomere measurement. We measured relative telomere length (RTL) for all samples using a quantitative PCR (qPCR) assay of telomeres and a GAPDH control gene, following Bebbington et al. (2016). Prior to qPCR, we used a random number generator to assign samples to qPCR plates, to ensure that no systematic bias could occur with regard to age, sex, cohort or ecological environment. Based on the distribution of observed cq values, we excluded outlier samples with extremely large cq values (cq values >25 and 26 were excluded for the telomere and GAPDH reactions respectively), which were assumed to be failed reactions.

For a large subset of birds we had longitudinal data, with two or more samples taken at different ages (*n* = 1,057 measurements from 402 birds). For these individuals we calculated the within‐individual change in RTL by subtracting RTL at time point *t* from RTL at time point *t* + 1 (hereafter ∆RTL, *n* = 655 measurements). Negative values of RTL reflect decreases in telomere length with age, while positive values reflect increases. Individuals were not always caught in the same month, but were generally caught within a 3‐month breeding season window.

### 2.3 Statistical analyses

We performed all statistical analyses using R version 3.2.2 (R Development Core Team, 2011). RTL was square root transformed to improve linear model fits, and we calculated mean values for samples with repeat measurements. We assessed repeatability of RTL using the rptR package.

We explored the cross‐sectional relationship between RTL and age among cohorts using linear mixed models (LMMs) carried out in the lme4 package (Bates, Maechler, Bolker, & Walker, 2014). Following a similar approach to Fairlie et al. (2016), we compared a selection of models fitting different relationships between RTL and age. We created models where the relationship between RTL and age was linear, quadratic, loglinear and where age was fitted as a factor. For each age term, we fitted additional models including hatch year (cohort) as a factor. All fitted models are included in Table 1. Note that we do not carry out full model selection or model averaging here, as our aim was to compare a set of specifically defined models. For random effects we included individual ID, catch year and qPCR plate ID. Models were compared using AIC with correction for finite sample size (AIC_c_; Hurvich & Tsai, 1989).

**Table 1:** Cross‐sectional (a) and longitudinal (b) telomere dynamics and age in Seychelles warbler cohorts. Linear mixed models were created with relative telomere length (RTL) (a) or ∆RTL (b) as the response variable, and different measures of age, along with cohort ID, were included as explanatory variables (see Section 2 for details). Models are ranked by AIC_c_, with best models at the top of the table

Model	*df*	AIC_c_	Delta AIC_c_	Weight
(a)				
Cohort + Age (log)	27	−1,062.782	0	1
Age (quadratic) + Age (linear) + Cohort	28	−1,039.504	23.278	0
Age (linear) + Cohort	27	−1,035.072	27.71	0
Age (log)	6	−1,034.942	27.84	0
Cohort + Age (factor)	41	−1,027.498	35.284	0
Age (quadratic) + Age (linear)	7	−1,013.793	48.989	0
Age (linear)	6	−1,006.873	55.909	0
Age (factor)	20	−1,004.885	57.897	0
Cohort	26	−1,000.037	62.745	0
Null model	5	−989.909	72.873	0
(b)				
Delta age (log) + Mean age	7	−370.124	0	0.459
Delta age (linear) + Mean age	7	−368.331	1.792	0.187
Cohort + Delta age (log) + Mean age	28	−367.567	2.556	0.128
Delta age (linear) + Delta age (quadratic) + Mean age	8	−366.53	3.594	0.076
Cohort + Delta age (linear) + Mean age	28	−366.467	3.657	0.074
Mean age	6	−365.397	4.726	0.043
Cohort + Delta age (linear) + Delta age (quadratic) + Mean age	29	−364.538	5.586	0.028
Cohort + Mean age	27	−360.94	9.184	0.005

Using the longitudinal data, we then tested how telomeres change with age in individuals, using LMMs of RTL as a response and ∆age (a longitudinal measure based on within‐subject centring; van de Pol & Wright, 2009) as an explanatory variable. We calculated ∆age using log and polynomial transformed age data, and carried out model selection as above, with the exception that we did not model ∆age as a factor (due to a lack of discreet groupings), and mean age was also included in models to partition within‐individual vs. cross‐sectional effects (van de Pol & Wright, 2009). We also excluded the qPCR plate ID random effect from the longitudinal analyses, as each longitudinal measurement was obtained from reactions run on separate plates.

We used two approaches to determine individual‐level consistency in RTL. We first calculated individual‐level repeatability in RTL by dividing the random variance explained by individual ID by the total random variance, in a model of that accounted for age and cohort effects. Second, we constructed a LMM with RTL at time *t* + 1 as the response variable, RTL at time *t* and age at time *t* as fixed effects, and individual ID and cohort as random effects. We estimated the slope of the relationship between within‐individual telomere measurements, as well as the variance explained, by calculating the marginal *R* ^2^ (Nakagawa & Schielzeth, 2013) of the model.

When examining the distribution of longitudinal telomere changes we observed some increases in telomere length with age in individuals. We therefore repeated the qPCR on a large number of samples, using completely separate reactions run on separate plates. We used these repeat measurements to test whether these increases could be explained by measurement error. We calculated the change in RTL between pairs of repeat measurements within the same samples (hereafter ∆RTL_sample_; *N* = 422 pairs of measurements from 293 birds) in exactly the same way as for across samples (hereafter ∆RTL_individual_). To test whether greater changes in RTL were observed among individuals compared to among repeat samples, we compared the variance in ∆RTL_sample_ and ∆RTL_individual_ using a Levene's test. Then, to separately test whether the extent of telomere increases and decreases within individuals were greater than expected by measurement error, we split ∆RTL measurements into groups in which RTL decreased (∆RTL < 0) and increased ∆RTL (∆RTL > 0), and tested whether ∆RTL_individual_ values were significantly different from ∆RTL_sample_ values, using Wilcoxon tests.

We also tested whether consistent telomere lengthening occurred across our dataset using a modified version of the approach developed by Simons et al. (2014). Briefly, this approach utilises samples with at least three telomere measurements to compare residual variance in telomere change over time with the overall change in telomere length between the first and last telomere measurements (Simons et al., 2014). If, in samples that increase in length, the overall increase in telomere length exceeds the residual variance, then telomere lengthening cannot be explained by error (Simons et al., 2014). If, on the other hand, increases in telomere length are due to measurement error, within‐individual residual variance in telomere length is expected to be similar to overall observed increases in telomere length.

We used LMMs to explore how variation in environmental and social conditions influenced telomere length and dynamics within cohorts. We first created a full model with RTL as a response variable, alongside the following explanatory variables: log age (based on the RTL and age analysis; see Section 3), tarsus length at capture, body mass at capture, sex, insect abundance in sampling season, territory quality in sampling season, island‐wide population density in sampling season (an annual measure estimated from the summer breeding census), territory group size in sampling season and the number of helping subordinate birds present in the territory in the sampling season. The random effects structure was informed by the analysis of telomere dynamics and age (see Section 3): we included individual ID, qPCR plate ID, cohort ID and a random slope of log age among cohorts (to allow the effect of age on RTL to vary among cohorts). We report model estimates and confidence intervals for all effects included in the full model. We also calculated marginal *R* ^2^ (incorporating only fixed effects; Nakagawa & Schielzeth, 2013) and conditional *R* ^2^ (incorporating fixed and random effects; Johnson, 2014) to assess the explanatory power of these models. As a complementary approach, we also performed model averaging, using the muMin package in R (Bartoń, 2012). Model selection was performed using the full model described above. A top model set was then defined, containing all models with AIC_c_ ≤ 6 compared with the best supported model (Burnham, Anderson, & Huyvaert, 2011). We report model‐averaged coefficients, confidence intervals and “relative importance,” which reflects the relative weights of each predictor variable across the top model set.

For individuals with longitudinal data we repeated the above analyses of telomere dynamics, replacing telomere length with ∆RTL_individual_ as the response variable, and including the environmental/social explanatory variables from the first of the two sampling points. We excluded the qPCR plate ID random effect from this analysis (see above), and excluded the cohort ID random effect, as longitudinal telomere dynamics did not differ among cohorts; see Section 3.

## 3 RESULTS

We measured telomere lengths using a total of 1,808 unique samples from juvenile and adult Seychelles warblers from 22 cohorts born between 1993 and 2014 (Table S1). Efficiencies (*M* ± *SD*) of our telomere and GAPDH reactions were 1.78 ± 0.05 and 1.92 ± 0.04 respectively. Intra‐plate repeatability was 0.74 (CI = 0.74, 0.75) and 0.73 (CI = 0.71, 0.74) for the GAPDH and Telomere Cq values respectively. Inter‐plate repeatability of RTL, based on 422 samples measured at least twice at different time points, was 0.68 (CI = 0.65, 0.70). Using samples taken from adults greater than 1 year old, we checked whether RTL was related to sample storage time, and found no evidence of such a relationship (estimate = −0.002, CIs = −0.007, 0.002).

### 3.1 Telomere dynamics and age among cohorts

**Figure 1:**
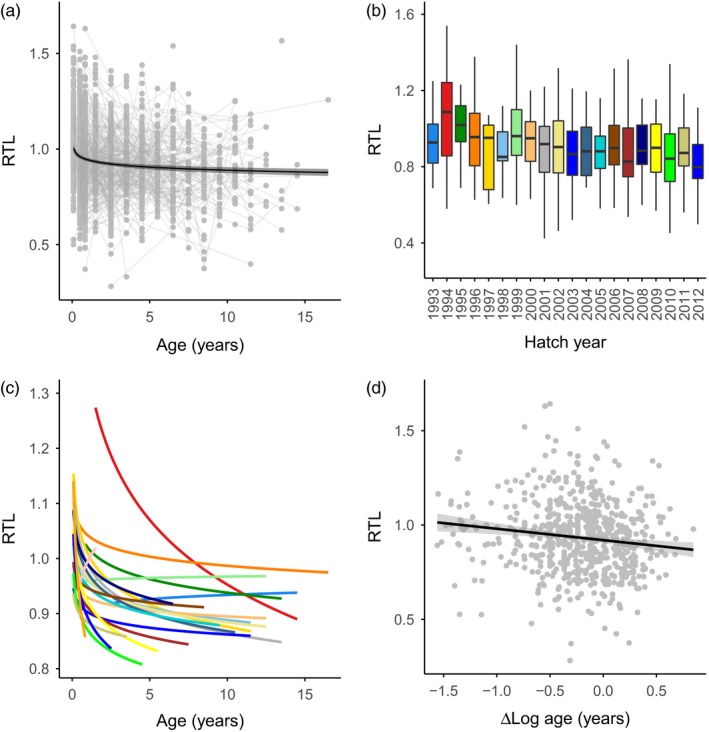
Telomere dynamics in relation to age in Seychelles warbler cohorts. (a) Relative telomere length (RTL) and age across all individuals. Points and connecting thin grey lines represent individual samples and birds respectively. The thick line and shaded area represent the fitted values and 95% confidence limits of a linear regression of RTL and log‐transformed age. (b) Boxplot of variation in RTL among juvenile individuals from all cohorts. (c) RTL and age among cohorts. Lines represent fitted values from a linear regression of RTL and log‐transformed age, and colours correspond to b. (d) RTL in relation to and ∆Log age (i.e. within‐individual variation in log age)

We first tested how RTL was related to age among cohorts using a model selection approach. The top model contained cohort ID and a loglinear relationship between RTL and age (Table 1a). All other models fitted the data much less well (∆AIC_c_ > 20; Table 1a). The loglinear relationship between RTL and age could be seen clearly in the raw data; RTL decreased with age (estimate = −0.070, CIs = −0.085, −0.054), with the greatest decrease occurring in the first year of life (Figure 1a). There was substantial variation in RTL among cohorts, with no obvious trend over time (Figure 1b). There was a negative relationship between RTL and log age in 21 of the 22 cohorts, but the slope of the relationship varied substantially among cohorts (Figure 1c). To test whether this variation was significant we fitted a model including the log age × cohort interaction term, and found that this was a marginally better fit than a model including only main effects (∆AIC_c_ = 11.32). In the 1 year in which RTL increased with age (2013), 17 of the 18 birds sampled were fledglings or subadults, suggesting that the observed pattern was an artefact of the sampling in this season (i.e. a lack of variation in age among sampled birds), rather than a real relationship.

A within‐individual analysis of RTL and age revealed that the top model explaining RTL contained ∆log age, which reflects within‐individual changes in log‐transformed age (Table 1b). Models including cohort ID were substantially poorer fits than a model only containing age (Table 1b). RTL decreased with ∆log age (estimate = −0.052, CIs = −0.085, −0.018), confirming that within‐individual telomere shortening occurs across the Seychelles warbler dataset. Furthermore, we found no evidence that within and between individual slopes of telomere shortening varied (estimate = −0.003, CIs = −0.008, 0.003), suggesting that there was no difference between cross‐sectional and longitudinal telomere shortening with age (see van de Pol & Wright, 2009).

**Figure 2:**
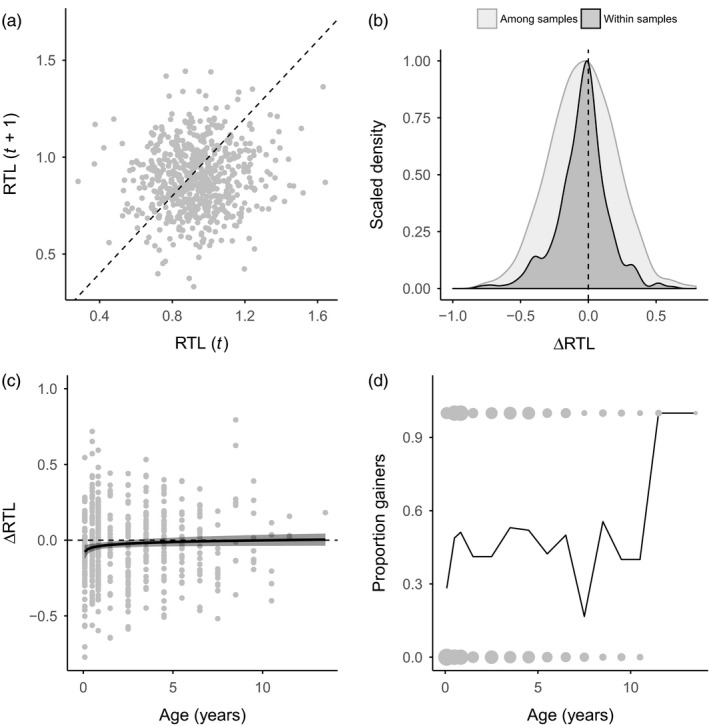
Longitudinal telomere dynamics in the Seychelles warbler. (a) Variation in relative telomere length (RTL) within individuals sampled at different time points. The dotted line represents parity, and thus points above and below the line represent increases and decreases in RTL respectively. (b) Scaled density plots of repeated RTL measurements among individual samples, and among different samples taken from the same individual. Areas of the density plot to the left of the dotted line represent decreases in RTL, while areas to the right represent increases. (c) ∆RTL in relation to age in pairs of samples taken within 2 years. Black line and shaded area represent fitted values and 95% confidence limits from a linear regression of RTL and log‐transformed age. (d) Probability of telomere lengthening occurring in relation to age. Points at zero and one represent pairs of samples where RTL has decreased and increased, respectively, with point size scaled by the number of overlapping values. The black line represents the proportion of samples in which increases in RTL where observed at each age category

Individual repeatability in RTL was 0.068, meaning that 7% of variance in RTL could be explained by within‐individual consistency. Accordingly, there was a positive correlation between RTL measured from different samples taken at different time points during an individual's life (Figure 2a), but this was very weak (marginal *R* ^2^ = 0.01), and not significant (estimate = 0.066, CIs = −0.006, 0.137).

Although both cross‐sectional and longitudinal data indicated a general trend of telomere shortening with age, we found that RTL—measured across two samples taken from the same individuals over time—increased with age in 44% of our 655 ∆RTL_individual_ measurements (Figure 2a). To test whether increases in telomere length in our dataset could be explained by measurement error, we compared variance in telomere length among repeat measurements of the same samples to the variance observed among different samples of the same individual. We found significantly higher variance in telomere length over individual lifetimes compared to among sample replicates (Levene's test: *F* = 43.63; *p* < .001; Figure 2b). Splitting the longitudinal data into instances of decreasing (i.e. ∆RTL < 0) and increasing (i.e. ∆RTL > 0) telomere length revealed that not only did we observe significantly greater decrease in RTL within individuals compared to within samples (Wilcoxon test: *p* < .001) but also a significantly greater increase (*p* < .001; Figure 2b).

To better understand how longitudinal telomere dynamics vary with age, we examined patterns of short‐term telomere change, including only pairs of samples taken within 2 years of each other. We found that the likelihood of telomere lengthening increased with log age (GLMM with lengthened yes/no as binomial response; estimate = 0.296, CIs = 0.005, 0.588). Increases in telomere length were most likely to be observed shortly after the juvenile period, at around 4 years of age, and later in life (although sample sizes for older birds are much smaller; Figure 2c, d).

Using the approach outlined by Simons et al. we tested whether overall increases in RTL over life spans could be detected statistically in our dataset. We found no evidence that this was the case: overall increases in RTL within individuals did not exceed residual variance; in fact, residual variance in RTL was significantly greater than observed RTL increases over life spans (*p* = .02). This suggests that increases in RTL within individuals are sporadic, and not consistent over individual life spans.

### 3.2 Telomere dynamics and the environment

**Figure 3:**
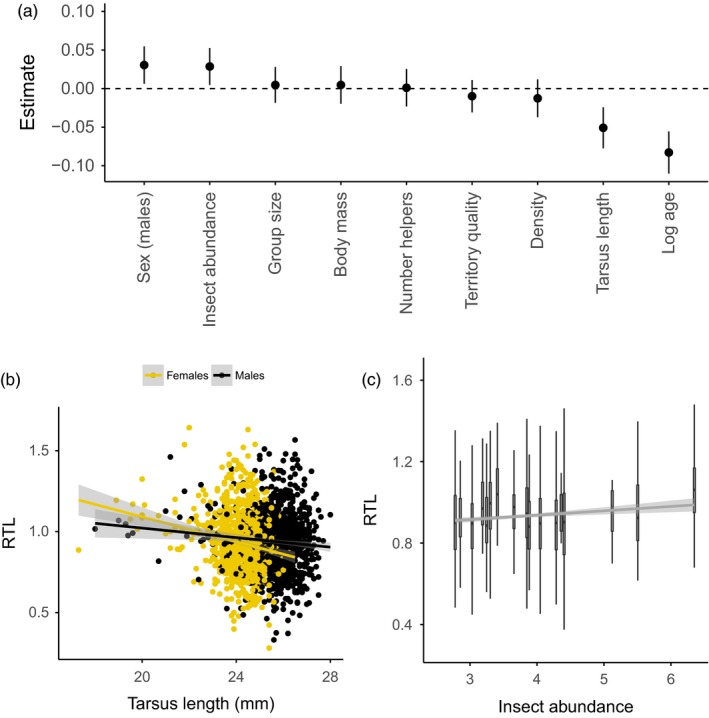
Telomere length in relation to the social and ecological environment in the Seychelles warbler. (a) Estimates and 95% confidence intervals for all explanatory variables fitted in a linear mixed model (see methods for details). (b) Relative telomere length (RTL) in relation to tarsus length and sex. (c) RTL in relation to variation in annual food availability. Lines and shaded areas represent the fitted values and 95% confidence limits from linear regressions [Colour figure can be viewed at wileyonlinelibrary.com]

In addition to age, RTL was associated with tarsus length, sex and insect abundance (Figure 3a). RTL was negatively related to tarsus length and males had longer telomeres than females (Figure 3b), while insect abundance was positively related to RTL (Figure 3c). The full model was weak in terms of explanatory power of fixed effects (marginal *R* ^2^ = 0.07), although including the random effect terms increased this substantially (conditional *R* ^2^ = 0.22). The model averaging approach yielded qualitatively identical results to the full LMM, with the same explanatory variables “significant” in terms of being retained in top models, and having model‐averaged confidence intervals not overlapping zero (Table S2; Figure S2). One interesting finding from the model selection was that sex only appeared in top models where tarsus length was also present (Table S2). In accordance with this, when tarsus length was removed from the full model sex was no longer significant (estimate = 0.008, CIs = −0.014, 0.030), and a sex × tarsus interaction was significant when included (estimate = 0.021, CIs = 0.002, 0.040); RTL decreased with tarsus length in both sexes, but this decrease was stronger in females (Figure 3b). No social or ecological environmental variables were significant predictors of ∆RTL using the full model approach (Table S3). Using model selection, we found that the top model explaining ∆RTL contained age and population density (Table S4). ∆RTL was positively related to age, consistent with telomere shortening being highest in early life, and negatively related to population density; however, in both instances model‐averaged confidence intervals overlapped zero (Figure S3).

## 4 DISCUSSION

Here, we use a long‐term, multi‐cohort dataset to assess lifelong telomere dynamics and the relationship between these and spatio‐temporal variation in the ecological environment in a contained population of Seychelles warblers. We found that telomere length decreases with age, and that this decrease is greatest very early in life. Telomere length decreased with age in almost all of the 22 cohorts studied, but telomere length varied substantially among cohorts. Despite an overall pattern of telomere shortening with age in the Seychelles warbler, we found evidence of within‐individual increases in telomere length, and that the extent of these increases could not be explained solely by qPCR measurement error. Finally, we found that telomeres are related to tarsus length in a sex‐specific manner, and that telomere length is positively associated with temporal fluctuations in food availability.

Our study adds to the substantial body of literature from humans and wild animals showing that telomere length decreases with age, and that this decrease is most rapid in early life. Rapid telomere shortening in early life occurs as a consequence of the much more rapid rate of cellular division that occurs not only during the growth phase but also perhaps higher levels of cellular stress during development (e.g. Frenck et al., 1998; Haussmann, Vleck, & Nisbet, 2003; Heidinger et al., 2012). Because we are rarely able to sample Seychelles warblers more than once in the nest, our system is not the best suited for looking in detail at the reasons behind telomere shortening during the growth phase. In contrast, we have good longitudinal and cross‐sectional sampling from across individual life spans, and using these data we have shown that, despite an overall trend for shortening, telomere length both increased and decreased, especially after the juvenile period. Importantly, these increases were observed in longitudinal as well as cross‐sectional data, indicating that selective disappearance of individuals with shorter telomeres is not sufficient to explain this pattern. Longitudinal increases in measured telomere length have been observed in humans and wild animals (Fairlie et al., 2016; Hoelzl, Cornils, Smith, Moodley, & Ruf, 2016; Hoelzl, Smith, et al. 2016; Kotrschal, Ilmonen, & Penn, 2007; Steenstrup et al., 2013). The most commonly invoked explanation for increases in telomere length is measurement error, which can be a particular problem in qPCR‐based telomere studies (Nussey et al., 2014; Steenstrup et al., 2013; Verhulst et al., 2015). However, recent modelling work suggests that longitudinal telomere dynamics in humans are indeed consistent with instances of lengthening, and that dismissing apparent telomere lengthening solely as a measurement error is “too strong” without additional data (Bateson & Nettle, 2016). Here, we explicitly compare intra‐individual variation among samples with variation among sample replicates, on a large scale. Our results suggest that despite the substantial levels of qPCR measurement error in our study, error alone cannot explain observed increases in RTL observed within individuals.

Increases in telomere length were not consistent over individual life spans, but occurred in bouts, against a backdrop of overall lifelong telomere shortening. This is consistent with recent findings in edible dormice *Glis glis*, in which telomere elongation was observed only later in life (Hoelzl, Smith, et al. 2016). Consistent with a pattern of sporadic changes in telomere length with age, we found that within‐individual telomere measurements were only weakly correlated. Although some of this low within‐individual repeatability will occur due to measurement error, our within‐sample repeatability was still much higher than our within‐individual repeatability. Such a low value of within‐individual repeatability in telomere length is in contrast to other avian studies in which within‐individual telomere length measurements were highly consistent, and individual‐level telomere shortening occurred throughout the juvenile period and into adulthood (Boonekamp, Mulder, Salomons, Dijkstra, & Verhulst, 2014; Heidinger et al., 2012). However, the lifelong telomere dynamics found in Seychelles warblers are strikingly similar to those found in Soay sheep (Fairlie et al., 2016). This discrepancy in results may be because in our study, and that of Fairlie et al. (2016), individuals were born and reared in the wild, as opposed to in nestbox or laboratory conditions. Alternatively it may be because our longitudinal telomere measurements have been taken over longer time periods.

The finding that increases in telomere length may be sporadic and overlaid on an overall pattern of shortening with age is an important point when assessing the occurrence of telomere lengthening. Previously described approaches to distinguish telomere elongation from measurement error, based on assumptions about follow‐up time between measurements (Steenstrup et al., 2013), or based on measuring variance among measurements (Simons et al., 2014), assume that telomere elongation within individuals is consistent over time. Our data, and that of others (Fairlie et al., 2016; Hoelzl, Smith, et al. 2016) suggest that this is not the case. Such inconsistent changes in telomere length over life spans could occur due to changes in the cellular composition of the blood within individual samples, due to variation in the presence of interstitial telomeric sequence, or due to the actual elongation of telomeres (Blackburn et al., 1989). Determining the mechanism of these changes is essential for how we view telomeres as biomarkers of costs. For example, if telomeres can be lengthened in response to improvements in environmental conditions, this would suggest that they reflect short‐ to medium‐term costs, rather than the cumulative costs that an individual has faced over its life span (Bateson, 2016). It is clear that telomeres can be a marker of long‐term as well as short‐term costs, as telomere length has been associated with both survival and life span in wild populations (Barrett et al., 2013; Stier et al., 2015), but we do not yet know how biologically meaningful within‐individual fluctuations in telomere length are. New research is therefore required to determine when and why telomere length increases within individuals, so that biologically informed hypotheses about the nature of telomeres as biomarkers in wild populations.

Measurement of cohorts across seasons or years is required if we are to understand how the environment impacts telomere dynamics. Although a few studies have shown that temporal variation in telomere dynamics occurs in natural populations, these have been limited in the number of seasons they cover (Fairlie et al., 2016; Mizutani, Tomita, Niizuma, & Yoda, 2013; Watson, Bolton, & Monaghan, 2015). Other studies have found cohort effects but not discussed them in an ecological context (Becker et al., 2015; Stier et al., 2014). One problem with studying cohort effects is that it can be difficult to tease apart true cohort effects from effects that may arise if samples degrade with storage time, and/or batch effects in telomere assays, although neither of these factors were a problem in our study. Indeed, the long‐term Seychelles warbler dataset has allowed us to show that temporal variation in telomere dynamics can occur over substantial time periods. Our data suggest that conditions during the hatch year are a very important factor in shaping telomere dynamics throughout life span. Thus, our findings suggest that the telomere dynamics of a population at a given point in time represent a snapshot of a temporally varying process. Research of telomere dynamics within and across multiple cohorts and populations will enable us to better understand how and why population‐level telomere dynamics vary over space and time.

We found that temporal variation in insect prey availability was positively related to telomere length. This is consistent with the strong cohort effects we found, and suggests that temporal variation in environmental conditions may be a key driver of costs in the Seychelles warbler. Although the environmental conditions on Cousin Island are relatively benign in comparison to other island systems (e.g. Coulson et al., 2001), substantial annual variation in rainfall does occur, with associated changes in insect abundance (Komdeur, 1996), and it appears that this confers a cost—in terms of intrinsic biological condition—to Seychelles warblers. Our results concur with other studies which show that early life conditions/food availability can have very significant and long‐term impacts on telomere length (and intrinsic biological condition) in captive and wild animals (e.g. Nettle et al., 2015; Stier et al., 2014; Watson et al., 2015).

We also found evidence for sex‐specific telomere dynamics: males had longer telomeres than females. Interestingly this sex difference interacts with tarsus length: telomere length was negatively correlated with tarsus length in both sexes, but this effect was stronger in females than males. If the sex‐dependent relationship between telomere and tarsus length was due to differential growth alone then we would expect the opposite pattern to that observed, as male Seychelles warblers are larger than females (Figure 3b). One possibility is that the environment imposes differential costs on males and females: a recent study in captive zebra finches found that manipulation of dietary nutrients had sex‐dependent effects on telomere dynamics (Noguera, Metcalfe, Boner, & Monaghan, 2015). Also worth noting is that the effect of telomere length on survival is strongest in male Seychelles warblers (Barrett et al., 2013), although comparative research suggests that the nature of the relationship between sex, telomeres and survival is not yet clear (Barrett & Richardson, 2011).

It is worth considering the fact that the social and ecological variables we tested here explained only a small proportion of the variance in RTL. Furthermore, some factors such as territory quality and social group size were not related to telomere dynamics when we may have expected them to be (Brouwer et al., 2012; Van de Crommenacker, Komdeur, & Richardson, 2011). Measurement error is clearly an issue in our study, and has almost certainly decreased the explanatory power of our models, and elevated levels of Type II error. The low repeatability we observed is the product of (1) lower levels of efficiency in the telomere qPCR reaction than we would have liked and (2) the long‐term nature of the study. Samples for this study were run over a period of up to 6 years, during which reagents, consumables and personnel all change. Our repeatability estimate includes samples that were run several years apart, and reflect all the sources of error that accumulated over that time. Compared with a set‐up where a small amount of samples are all run at the same time, it is unsurprising that we have a higher error rate—indeed, in our own studies where we have run all the samples within a short timeframe we have observed higher repeatability (Barrett et al., 2012; Bebbington et al., 2016). Techniques for measuring telomere length with a greater degree of precision are likely to be proven helpful in future long‐term ecological studies of telomere dynamics (Nussey et al., 2014), and discussions are now required on how to best optimise measuring telomere length for long‐term studies. A central issue to resolve is how best to balance the trade‐off between obtaining precise telomere measurements, and utilising the large sample sizes necessary for ecological study.

While sampling error is a problem in our study, we are confident that because of our study design, and plate randomisation in particular, sampling error is highly unlikely to have resulted in a high false positive rate. This is clearly a problem that needs to be considered, however, when designing long‐term studies of telomere dynamics. And, clearly, sampling error is not the only factor contributing to the unexplained variance in telomere length in our study. It should always be borne in mind that, in any system, unmeasured environmental and genetic variables will contribute to unexplained variance in telomere dynamics. A key question to be addressed is the extent to which RTL, especially in early life, reflects inheritance and parental effects (e.g. Asghar, Bensch, Tarka, Hansson, & Hasselquist, 2014; Becker et al., 2015; Heidinger et al., 2016). For example, parental age and quality may key variables that impact the telomere dynamics of offspring in the Seychelles warbler, and will be addressed in future studies. Long‐term ecological study systems are uniquely suited for addressing such questions in natural systems (Clutton‐Brock & Sheldon, 2010). To gain a full understanding of telomere dynamics in natural systems, long‐term studies combining ecological and genetic data will be required from a range of species.

## AUTHORS’ CONTRIBUTIONS

D.S.R., H.L.D., J.K. and T.B. manage the long‐term Seychelles warbler project. D.S.R. conceived and obtained funding for the telomere research. E.A.F. and K.B. performed the molecular work. L.G.S. processed the telomere data, with input from E.A.F., K.B., M.H., H.L.D. and D.S.R. L.G.S. analysed the data and wrote the manuscript, with input from D.S.R. and all authors.

## DATA ACCESSIBILITY

All data and scripts required to reproduce the manuscript, figures and analyses are available on GitHub https://doi.org/10.5281/zenodo.835844 (Spurgin et al. 2017).
