# Supplementary material for: Spatio‐temporal variation in lifelong telomere dynamics in a long‐term ecological study
Source: J Anim Ecol. 2017 Sep 28;87(1):187–98. doi: 10.1111/1365-2656.12741 (PMC5765431; doi:10.1111/1365-2656.12741)
Supplement: Supplementary file 1 [file JANE-87-187-s001.docx]

# Supplementary information for Spurgin *et al.* "Spatiotemporal variation in lifelong telomere dynamics in a long-term ecological study"

**Table S1** Cohort sizes for each hatch year of the Seychelles warbler dataset used in this study. Note that cohort sizes on Cousin are typically small due to the saturated nature of the population.

| Cohort | N |
| --- | --- |
| 1993 | 24 |
| 1994 | 18 |
| 1995 | 11 |
| 1996 | 46 |
| 1997 | 25 |
| 1998 | 22 |
| 1999 | 45 |
| 2000 | 33 |
| 2001 | 46 |
| 2002 | 73 |
| 2003 | 46 |
| 2004 | 44 |
| 2005 | 90 |
| 2006 | 88 |
| 2007 | 61 |
| 2008 | 47 |
| 2009 | 73 |
| 2010 | 75 |
| 2011 | 62 |
| 2012 | 45 |
| 2013 | 13 |
| 2014 | 53 |

**Table S2** Model selection table for analysis of factors affecting early life telomere length in juvenile Seychelles warblers. Only models that remained in the top model set for model averaging (AICc $\leq$ 6 compared to the top model) are included here.

| Model | df | AICc | Delta AICc | Weight |
| --- | --- | --- | --- | --- |
| Sex (males) + Insect abundance + Log age + Tarsus | 11 | -875.68 | 0.00 | 0.16 |
| Sex (males) + Density + Insect abundance + Log age + Tarsus | 12 | -874.18 | 1.51 | 0.07 |
| Sex (males) + Body mass + Insect abundance + Log age + Tarsus | 12 | -873.73 | 1.95 | 0.06 |
| Sex (males) + Group Size + Insect abundance + Log age + Tarsus | 12 | -873.73 | 1.95 | 0.06 |
| Helpers + Sex (males) + Insect abundance + Log age + Tarsus | 12 | -873.72 | 1.96 | 0.06 |
| Sex (males) + Log age + Tarsus | 10 | -873.56 | 2.12 | 0.05 |
| Sex (males) + Density + Insect abundance + Log age + Tarsus + Territory quality | 13 | -872.84 | 2.84 | 0.04 |
| Sex (males) + Density + Group Size + Insect abundance + Log age + Tarsus | 13 | -872.27 | 3.41 | 0.03 |
| Helpers + Sex (males) + Density + Insect abundance + Log age + Tarsus | 13 | -872.25 | 3.43 | 0.03 |
| Sex (males) + Body mass + Density + Insect abundance + Log age + Tarsus | 13 | -872.19 | 3.49 | 0.03 |
| Sex (males) + Group Size + Insect abundance + Log age + Tarsus + Territory quality | 13 | -872.06 | 3.63 | 0.03 |
| Sex (males) + Body mass + Insect abundance + Log age + Tarsus + Territory quality | 13 | -872.05 | 3.63 | 0.03 |
| Helpers + Sex (males) + Insect abundance + Log age + Tarsus + Territory quality | 13 | -872.01 | 3.67 | 0.02 |
| Sex (males) + Density + Log age + Tarsus | 11 | -871.84 | 3.84 | 0.02 |
| Sex (males) + Body mass + Group Size + Insect abundance + Log age + Tarsus | 13 | -871.81 | 3.87 | 0.02 |
| Helpers + Sex (males) + Body mass + Insect abundance + Log age + Tarsus | 13 | -871.78 | 3.90 | 0.02 |
| Sex (males) + Log age + Tarsus + Territory quality | 11 | -871.78 | 3.90 | 0.02 |
| Helpers + Sex (males) + Group Size + Insect abundance + Log age + Tarsus | 13 | -871.71 | 3.97 | 0.02 |
| Helpers + Sex (males) + Log age + Tarsus | 11 | -871.61 | 4.08 | 0.02 |
| Sex (males) + Group Size + Log age + Tarsus | 11 | -871.60 | 4.09 | 0.02 |
| Sex (males) + Body mass + Log age + Tarsus | 11 | -871.53 | 4.15 | 0.02 |
| Sex (males) + Density + Group Size + Insect abundance + Log age + Tarsus + Territory quality | 14 | -871.03 | 4.65 | 0.02 |
| Helpers + Sex (males) + Density + Insect abundance + Log age + Tarsus + Territory quality | 14 | -870.93 | 4.75 | 0.01 |
| Insect abundance + Log age + Tarsus | 10 | -870.48 | 5.21 | 0.01 |
| Sex (males) + Body mass + Density + Group Size + Insect abundance + Log age + Tarsus | 14 | -870.33 | 5.35 | 0.01 |
| Sex (males) + Density + Log age + Tarsus + Territory quality | 12 | -870.29 | 5.39 | 0.01 |
| Helpers + Sex (males) + Body mass + Density + Insect abundance + Log age + Tarsus | 14 | -870.28 | 5.40 | 0.01 |
| Helpers + Sex (males) + Density + Group Size + Insect abundance + Log age + Tarsus | 14 | -870.26 | 5.42 | 0.01 |
| Sex (males) + Body mass + Group Size + Insect abundance + Log age + Tarsus + Territory quality | 14 | -870.18 | 5.50 | 0.01 |
| Helpers + Sex (males) + Body mass + Insect abundance + Log age + Tarsus + Territory quality | 14 | -870.10 | 5.58 | 0.01 |
| Helpers + Sex (males) + Group Size + Insect abundance + Log age + Tarsus + Territory quality | 14 | -870.02 | 5.66 | 0.01 |
| Helpers + Sex (males) + Density + Log age + Tarsus | 12 | -869.92 | 5.77 | 0.01 |
| Sex (males) + Density + Group Size + Log age + Tarsus | 12 | -869.91 | 5.77 | 0.01 |
| Sex (males) + Group Size + Log age + Tarsus + Territory quality | 12 | -869.84 | 5.84 | 0.01 |
| Helpers + Sex (males) + Log age + Tarsus + Territory quality | 12 | -869.82 | 5.86 | 0.01 |
| Sex (males) + Body mass + Density + Log age + Tarsus | 12 | -869.81 | 5.87 | 0.01 |
| Helpers + Sex (males) + Body mass + Group Size + Insect abundance + Log age + Tarsus | 14 | -869.79 | 5.89 | 0.01 |
| Sex (males) + Body mass + Log age + Tarsus + Territory quality | 12 | -869.75 | 5.93 | 0.01 |

**Table S3** Full model of factors affecting $\Delta$RTL in Seychelles warblers, using longitudinal data.

| Variable | Estimate | LCI | UCI |
| --- | --- | --- | --- |
| (Intercept) | -0.037 | -0.058 | -0.016 |
| Age | 0.032 | -0.012 | 0.076 |
| Tarsus length | 0.047 | -0.012 | 0.106 |
| Number helpers | 0.021 | -0.037 | 0.078 |
| Group size | -0.018 | -0.072 | 0.036 |
| Sex (males) | -0.032 | -0.090 | 0.026 |
| Body mass | 0.009 | -0.044 | 0.062 |
| Insect abundance | -0.021 | -0.066 | 0.024 |
| Density | -0.042 | -0.091 | 0.008 |
| Territory quality | 0.003 | -0.045 | 0.052 |

**Table S4** Model selection table for analysis of factors affecting $\Delta$RTL in Seychelles warblers, using longitudinal data. Only models that remained in the top model set for model averaging (AICc $\leq$ 6 compared to the top model) are included here.

| Model | df | AICc | Delta AICc | Weight |
| --- | --- | --- | --- | --- |
| Density + Log age | 5 | 10.32 | 0.00 | 0.03 |
| Density + Log age + Tarsus | 6 | 10.53 | 0.21 | 0.03 |
| Density + Tarsus | 5 | 11.63 | 1.31 | 0.02 |
| Body mass + Density + Log age | 6 | 11.69 | 1.37 | 0.02 |
| Sex (males) + Density + Log age + Tarsus | 7 | 11.75 | 1.43 | 0.02 |
| Density + Insect abundance + Log age + Tarsus | 7 | 11.88 | 1.56 | 0.02 |
| Density + Insect abundance + Log age | 6 | 11.93 | 1.61 | 0.02 |
| Log age + Tarsus | 5 | 12.02 | 1.70 | 0.01 |
| Log age | 4 | 12.03 | 1.71 | 0.01 |
| Helpers + Density + Log age | 6 | 12.20 | 1.88 | 0.01 |
| Density + Group size + Log age | 6 | 12.29 | 1.97 | 0.01 |
| Sex (males) + Density + Log age | 6 | 12.33 | 2.01 | 0.01 |
| Density + Log age + Territory quality | 6 | 12.35 | 2.03 | 0.01 |
| Sex (males) + Density + Tarsus | 6 | 12.43 | 2.11 | 0.01 |
| Density | 4 | 12.44 | 2.12 | 0.01 |
| Helpers + Density + Log age + Tarsus | 7 | 12.49 | 2.17 | 0.01 |
| Density + Group size + Log age + Tarsus | 7 | 12.52 | 2.20 | 0.01 |
| Body mass + Density + Log age + Tarsus | 7 | 12.57 | 2.24 | 0.01 |
| Density + Log age + Tarsus + Territory quality | 7 | 12.59 | 2.26 | 0.01 |
| Density + Insect abundance + Tarsus | 6 | 12.78 | 2.46 | 0.01 |
| Log age + Territory quality | 5 | 12.92 | 2.60 | 0.01 |
| Sex (males) + Density + Insect abundance + Log age + Tarsus | 8 | 13.04 | 2.72 | 0.01 |
| Sex (males) + Log age + Tarsus | 6 | 13.07 | 2.75 | 0.01 |
| Body mass + Density | 5 | 13.08 | 2.76 | 0.01 |
| Log age + Tarsus + Territory quality | 6 | 13.14 | 2.82 | 0.01 |
| Tarsus | 4 | 13.28 | 2.96 | 0.01 |
| Body mass + Density + Insect abundance + Log age | 7 | 13.29 | 2.96 | 0.01 |
| Density + Group size + Tarsus | 6 | 13.45 | 3.13 | 0.01 |
| Body mass + Log age | 5 | 13.46 | 3.14 | 0.01 |
| Sex (males) + Density + Insect abundance + Tarsus | 7 | 13.52 | 3.20 | 0.01 |
| Helpers + Body mass + Density + Log age | 7 | 13.54 | 3.21 | 0.01 |
| Body mass + Density + Tarsus | 6 | 13.56 | 3.24 | 0.01 |
| Helpers + Density + Tarsus | 6 | 13.60 | 3.28 | 0.01 |
| Sex (males) + Body mass + Density + Log age + Tarsus | 8 | 13.61 | 3.28 | 0.01 |
| Sex (males) + Body mass + Density + Log age | 7 | 13.65 | 3.33 | 0.01 |
| Density + Tarsus + Territory quality | 6 | 13.67 | 3.35 | 0.01 |
| Sex (males) + Density + Group size + Log age + Tarsus | 8 | 13.69 | 3.37 | 0.01 |
| Helpers + Sex (males) + Density + Log age + Tarsus | 8 | 13.72 | 3.39 | 0.01 |
| Body mass + Density + Log age + Territory quality | 7 | 13.73 | 3.41 | 0.01 |
| Body mass + Density + Group size + Log age | 7 | 13.73 | 3.41 | 0.01 |
| Group size + Log age | 5 | 13.78 | 3.45 | 0.01 |
| Helpers + Density + Insect abundance + Log age | 7 | 13.79 | 3.47 | 0.01 |
| Group size + Log age + Tarsus | 6 | 13.80 | 3.48 | 0.01 |
| Sex (males) + Density + Log age + Tarsus + Territory quality | 8 | 13.81 | 3.49 | 0.01 |
| Helpers + Density + Insect abundance + Log age + Tarsus | 8 | 13.82 | 3.50 | 0.01 |
| Helpers + Density + Group size + Log age | 7 | 13.85 | 3.52 | 0.01 |
| Sex (males) + Tarsus | 5 | 13.86 | 3.54 | 0.01 |
| Density + Group size + Insect abundance + Log age + Tarsus | 8 | 13.87 | 3.54 | 0.01 |
| Density + Group size + Insect abundance + Log age | 7 | 13.90 | 3.58 | 0.01 |
| Helpers + Log age | 5 | 13.91 | 3.59 | 0.01 |
| Density + Insect abundance + Log age + Tarsus + Territory quality | 8 | 13.92 | 3.60 | 0.01 |
| Body mass + Density + Insect abundance + Log age + Tarsus | 8 | 13.93 | 3.61 | 0.01 |
| Density + Insect abundance | 5 | 13.93 | 3.61 | 0.01 |
| Sex (males) + Density + Insect abundance + Log age | 7 | 13.94 | 3.62 | 0.01 |
| Density + Insect abundance + Log age + Territory quality | 7 | 13.94 | 3.62 | 0.01 |
| Helpers + Log age + Tarsus | 6 | 13.98 | 3.65 | 0.01 |
| Sex (males) + Body mass + Density + Tarsus | 7 | 14.00 | 3.68 | 0.01 |
| Insect abundance + Log age + Tarsus | 6 | 14.00 | 3.68 | 0.01 |
| Sex (males) + Log age | 5 | 14.04 | 3.72 | 0.01 |
| Body mass + Log age + Tarsus | 6 | 14.07 | 3.75 | 0.01 |
| Insect abundance + Log age | 5 | 14.07 | 3.75 | 0.01 |
| Sex (males) + Density + Group size + Tarsus | 7 | 14.17 | 3.85 | 0.01 |
| Density + Group size | 5 | 14.20 | 3.88 | 0.00 |
| Helpers + Sex (males) + Density + Log age | 7 | 14.23 | 3.91 | 0.00 |
| Helpers + Density + Group size + Log age + Tarsus | 8 | 14.24 | 3.92 | 0.00 |
| Helpers + Density + Log age + Territory quality | 7 | 14.25 | 3.92 | 0.00 |
| Sex (males) + Log age + Tarsus + Territory quality | 7 | 14.29 | 3.96 | 0.00 |
| Sex (males) + Density + Group size + Log age | 7 | 14.32 | 4.00 | 0.00 |
| Helpers + Density | 5 | 14.32 | 4.00 | 0.00 |
| Density + Group size + Log age + Territory quality | 7 | 14.32 | 4.00 | 0.00 |
| Body mass + Log age + Territory quality | 6 | 14.36 | 4.03 | 0.00 |
| Sex (males) + Density + Log age + Territory quality | 7 | 14.37 | 4.05 | 0.00 |
| Null model | 3 | 14.40 | 4.08 | 0.00 |
| Helpers + Sex (males) + Density + Tarsus | 7 | 14.41 | 4.09 | 0.00 |
| Sex (males) + Density | 5 | 14.42 | 4.10 | 0.00 |
| Sex (males) + Density + Tarsus + Territory quality | 7 | 14.48 | 4.15 | 0.00 |
| Density + Territory quality | 5 | 14.48 | 4.16 | 0.00 |
| Helpers + Body mass + Density + Log age + Tarsus | 8 | 14.52 | 4.20 | 0.00 |
| Helpers + Density + Log age + Tarsus + Territory quality | 8 | 14.55 | 4.23 | 0.00 |
| Tarsus + Territory quality | 5 | 14.56 | 4.24 | 0.00 |
| Body mass + Density + Insect abundance | 6 | 14.56 | 4.24 | 0.00 |
| Body mass + Density + Group size + Log age + Tarsus | 8 | 14.57 | 4.25 | 0.00 |
| Density + Group size + Log age + Tarsus + Territory quality | 8 | 14.58 | 4.26 | 0.00 |
| Density + Group size + Insect abundance + Tarsus | 7 | 14.60 | 4.28 | 0.00 |
| Body mass + Density + Log age + Tarsus + Territory quality | 8 | 14.63 | 4.31 | 0.00 |
| Group size + Log age + Territory quality | 6 | 14.70 | 4.38 | 0.00 |
| Helpers + Density + Insect abundance + Tarsus | 7 | 14.73 | 4.41 | 0.00 |
| Sex (males) + Group size + Log age + Tarsus | 7 | 14.75 | 4.43 | 0.00 |
| Body mass + Density + Insect abundance + Tarsus | 7 | 14.76 | 4.43 | 0.00 |
| Group size + Tarsus | 5 | 14.77 | 4.45 | 0.00 |
| Density + Insect abundance + Tarsus + Territory quality | 7 | 14.84 | 4.51 | 0.00 |
| Helpers + Log age + Territory quality | 6 | 14.85 | 4.53 | 0.00 |
| Insect abundance + Log age + Territory quality | 6 | 14.85 | 4.53 | 0.00 |
| Helpers + Body mass + Density | 6 | 14.91 | 4.59 | 0.00 |
| Sex (males) + Body mass + Density | 6 | 14.91 | 4.59 | 0.00 |
| Insect abundance + Log age + Tarsus + Territory quality | 7 | 14.93 | 4.61 | 0.00 |
| Sex (males) + Body mass + Density + Insect abundance + Log age + Tarsus | 9 | 14.94 | 4.62 | 0.00 |
| Group size + Log age + Tarsus + Territory quality | 7 | 14.94 | 4.62 | 0.00 |
| Sex (males) + Log age + Territory quality | 6 | 14.95 | 4.63 | 0.00 |
| Sex (males) + Density + Group size + Insect abundance + Log age + Tarsus | 9 | 14.97 | 4.65 | 0.00 |
| Sex (males) + Body mass + Log age + Tarsus | 7 | 14.98 | 4.65 | 0.00 |
| Helpers + Sex (males) + Density + Insect abundance + Log age + Tarsus | 9 | 14.99 | 4.67 | 0.00 |
| Helpers + Group size + Log age | 6 | 15.02 | 4.70 | 0.00 |
| Sex (males) + Insect abundance + Log age + Tarsus | 7 | 15.02 | 4.70 | 0.00 |
| Helpers + Density + Group size + Tarsus | 7 | 15.02 | 4.70 | 0.00 |
| Helpers + Sex (males) + Log age + Tarsus | 7 | 15.03 | 4.71 | 0.00 |
| Body mass + Density + Group size | 6 | 15.05 | 4.73 | 0.00 |
| Sex (males) + Density + Insect abundance + Log age + Tarsus + Territory quality | 9 | 15.10 | 4.77 | 0.00 |
| Body mass | 4 | 15.11 | 4.79 | 0.00 |
| Helpers + Body mass + Density + Insect abundance + Log age | 8 | 15.11 | 4.79 | 0.00 |
| Helpers + Log age + Tarsus + Territory quality | 7 | 15.12 | 4.80 | 0.00 |
| Body mass + Density + Territory quality | 6 | 15.13 | 4.80 | 0.00 |
| Sex (males) + Body mass + Density + Insect abundance + Tarsus | 8 | 15.16 | 4.84 | 0.00 |
| Body mass + Log age + Tarsus + Territory quality | 7 | 15.18 | 4.86 | 0.00 |
| Insect abundance + Tarsus | 5 | 15.19 | 4.87 | 0.00 |
| Sex (males) + Group size + Tarsus | 6 | 15.23 | 4.91 | 0.00 |
| Sex (males) + Tarsus + Territory quality | 6 | 15.24 | 4.92 | 0.00 |
| Helpers + Tarsus | 5 | 15.24 | 4.92 | 0.00 |
| Sex (males) + Density + Group size + Insect abundance + Tarsus | 8 | 15.25 | 4.93 | 0.00 |
| Body mass + Tarsus | 5 | 15.26 | 4.94 | 0.00 |
| Sex (males) + Body mass + Density + Insect abundance + Log age | 8 | 15.28 | 4.96 | 0.00 |
| Helpers + Group size + Log age + Tarsus | 7 | 15.28 | 4.96 | 0.00 |
| Helpers + Body mass + Log age | 6 | 15.30 | 4.98 | 0.00 |
| Body mass + Density + Insect abundance + Log age + Territory quality | 8 | 15.30 | 4.98 | 0.00 |
| Body mass + Density + Group size + Insect abundance + Log age | 8 | 15.33 | 5.01 | 0.00 |
| Helpers + Sex (males) + Density + Group size + Log age + Tarsus | 9 | 15.35 | 5.03 | 0.00 |
| Body mass + Group size + Log age | 6 | 15.35 | 5.03 | 0.00 |
| Helpers + Body mass + Density + Group size + Log age | 8 | 15.37 | 5.05 | 0.00 |
| Helpers + Density + Group size + Insect abundance + Log age | 8 | 15.39 | 5.07 | 0.00 |
| Sex (males) + Body mass + Log age | 6 | 15.42 | 5.10 | 0.00 |
| Territory quality | 4 | 15.45 | 5.13 | 0.00 |
| Body mass + Density + Group size + Tarsus | 7 | 15.45 | 5.13 | 0.00 |
| Helpers + Sex (males) + Density + Insect abundance + Tarsus | 8 | 15.48 | 5.15 | 0.00 |
| Helpers + Sex (males) + Body mass + Density + Log age | 8 | 15.48 | 5.16 | 0.00 |
| Helpers + Density + Group size | 6 | 15.49 | 5.16 | 0.00 |
| Body mass + Insect abundance + Log age | 6 | 15.50 | 5.18 | 0.00 |
| Density + Group size + Tarsus + Territory quality | 7 | 15.51 | 5.18 | 0.00 |
| Sex (males) + Body mass + Tarsus | 6 | 15.51 | 5.19 | 0.00 |
| Helpers + Body mass + Density + Tarsus | 7 | 15.51 | 5.19 | 0.00 |
| Helpers + Density + Group size + Insect abundance + Log age + Tarsus | 9 | 15.53 | 5.21 | 0.00 |
| Helpers + Sex (males) + Body mass + Density + Log age + Tarsus | 9 | 15.54 | 5.22 | 0.00 |
| Sex (males) + Density + Insect abundance + Tarsus + Territory quality | 8 | 15.58 | 5.26 | 0.00 |
| Helpers + Body mass + Density + Log age + Territory quality | 8 | 15.59 | 5.27 | 0.00 |
| Sex (males) + Body mass + Density + Group size + Log age + Tarsus | 9 | 15.61 | 5.29 | 0.00 |
| Body mass + Density + Tarsus + Territory quality | 7 | 15.61 | 5.29 | 0.00 |
| Helpers + Density + Tarsus + Territory quality | 7 | 15.64 | 5.32 | 0.00 |
| Helpers + Sex (males) + Density + Group size + Tarsus | 8 | 15.65 | 5.33 | 0.00 |
| Sex (males) + Body mass + Density + Log age + Tarsus + Territory quality | 9 | 15.68 | 5.35 | 0.00 |
| Density + Group size + Insect abundance | 6 | 15.69 | 5.37 | 0.00 |
| Sex (males) + Body mass + Density + Log age + Territory quality | 8 | 15.70 | 5.38 | 0.00 |
| Sex (males) + Body mass + Density + Group size + Log age | 8 | 15.70 | 5.38 | 0.00 |
| Sex (males) + Insect abundance + Tarsus | 6 | 15.74 | 5.42 | 0.00 |
| Sex (males) + Density + Group size + Log age + Tarsus + Territory quality | 9 | 15.76 | 5.44 | 0.00 |
| Group size + Insect abundance + Log age + Tarsus | 7 | 15.77 | 5.45 | 0.00 |
| Body mass + Density + Group size + Log age + Territory quality | 8 | 15.78 | 5.45 | 0.00 |
| Group size | 4 | 15.78 | 5.46 | 0.00 |
| Helpers + Sex (males) + Density + Log age + Tarsus + Territory quality | 9 | 15.79 | 5.47 | 0.00 |
| Helpers + Density + Insect abundance | 6 | 15.79 | 5.47 | 0.00 |
| Sex (males) + Group size + Log age | 6 | 15.81 | 5.48 | 0.00 |
| Group size + Insect abundance + Log age | 6 | 15.81 | 5.49 | 0.00 |
| Helpers + Sex (males) + Density + Insect abundance + Log age | 8 | 15.82 | 5.49 | 0.00 |
| Helpers + Density + Insect abundance + Log age + Territory quality | 8 | 15.82 | 5.50 | 0.00 |
| Helpers + Sex (males) + Tarsus | 6 | 15.83 | 5.51 | 0.00 |
| Body mass + Group size + Log age + Tarsus | 7 | 15.85 | 5.53 | 0.00 |
| Helpers + Body mass + Density + Insect abundance + Log age + Tarsus | 9 | 15.87 | 5.55 | 0.00 |
| Helpers + Density + Insect abundance + Log age + Tarsus + Territory quality | 9 | 15.88 | 5.56 | 0.00 |
| Sex (males) + Body mass + Density + Group size + Tarsus | 8 | 15.89 | 5.57 | 0.00 |
| Helpers + Density + Group size + Log age + Territory quality | 8 | 15.89 | 5.57 | 0.00 |
| Helpers + Sex (males) + Density + Group size + Log age | 8 | 15.90 | 5.58 | 0.00 |
| Sex (males) + Density + Insect abundance | 6 | 15.91 | 5.58 | 0.00 |
| Density + Group size + Insect abundance + Log age + Territory quality | 8 | 15.91 | 5.59 | 0.00 |
| Density + Group size + Insect abundance + Log age + Tarsus + Territory quality | 9 | 15.91 | 5.59 | 0.00 |
| Sex (males) + Density + Group size + Insect abundance + Log age | 8 | 15.93 | 5.60 | 0.00 |
| Helpers + Sex (males) + Body mass + Density + Tarsus | 8 | 15.93 | 5.61 | 0.00 |
| Helpers + Sex (males) + Log age | 6 | 15.93 | 5.61 | 0.00 |
| Body mass + Density + Group size + Insect abundance + Log age + Tarsus | 9 | 15.94 | 5.61 | 0.00 |
| Helpers + Insect abundance + Log age | 6 | 15.95 | 5.62 | 0.00 |
| Helpers + Insect abundance + Log age + Tarsus | 7 | 15.95 | 5.63 | 0.00 |
| Sex (males) + Density + Insect abundance + Log age + Territory quality | 8 | 15.96 | 5.63 | 0.00 |
| Density + Insect abundance + Territory quality | 6 | 15.97 | 5.65 | 0.00 |
| Body mass + Density + Insect abundance + Log age + Tarsus + Territory quality | 9 | 15.98 | 5.66 | 0.00 |
| Sex (males) + Group size + Log age + Tarsus + Territory quality | 8 | 16.00 | 5.68 | 0.00 |
| Helpers + Body mass + Log age + Tarsus | 7 | 16.02 | 5.70 | 0.00 |
| Sex (males) + Insect abundance + Log age + Tarsus + Territory quality | 8 | 16.04 | 5.72 | 0.00 |
| Body mass + Insect abundance + Log age + Tarsus | 7 | 16.06 | 5.73 | 0.00 |
| Sex (males) + Body mass + Density + Tarsus + Territory quality | 8 | 16.06 | 5.73 | 0.00 |
| Helpers + Group size + Tarsus | 6 | 16.06 | 5.74 | 0.00 |
| Group size + Tarsus + Territory quality | 6 | 16.08 | 5.76 | 0.00 |
| Sex (males) + Insect abundance + Log age | 6 | 16.09 | 5.76 | 0.00 |
| Helpers + Group size + Log age + Territory quality | 7 | 16.09 | 5.77 | 0.00 |
| Helpers + Density + Group size + Insect abundance + Tarsus | 8 | 16.10 | 5.78 | 0.00 |
| Body mass + Territory quality | 5 | 16.14 | 5.82 | 0.00 |
| Helpers + Sex (males) + Group size + Log age + Tarsus | 8 | 16.15 | 5.82 | 0.00 |
| Sex (males) + Body mass + Log age + Tarsus + Territory quality | 8 | 16.17 | 5.85 | 0.00 |
| Sex (males) + Density + Group size | 6 | 16.21 | 5.89 | 0.00 |
| Sex (males) + Density + Group size + Tarsus + Territory quality | 8 | 16.23 | 5.91 | 0.00 |
| Density + Group size + Territory quality | 6 | 16.24 | 5.92 | 0.00 |
| Helpers + Body mass + Log age + Territory quality | 7 | 16.25 | 5.93 | 0.00 |
| Insect abundance + Tarsus + Territory quality | 6 | 16.26 | 5.94 | 0.00 |
| Body mass + Group size + Log age + Territory quality | 7 | 16.27 | 5.95 | 0.00 |
| Helpers | 4 | 16.27 | 5.95 | 0.00 |
| Helpers + Sex (males) + Log age + Tarsus + Territory quality | 8 | 16.28 | 5.96 | 0.00 |
| Helpers + Sex (males) + Density + Log age + Territory quality | 8 | 16.28 | 5.96 | 0.00 |
| Body mass + Insect abundance + Log age + Territory quality | 7 | 16.28 | 5.96 | 0.00 |
| Helpers + Body mass + Density + Group size + Log age + Tarsus | 9 | 16.31 | 5.99 | 0.00 |
| Helpers + Density + Group size + Log age + Tarsus + Territory quality | 9 | 16.31 | 5.99 | 0.00 |
| Sex (males) + Body mass + Log age + Territory quality | 7 | 16.32 | 5.99 | 0.00 |
| Helpers + Sex (males) + Density | 6 | 16.32 | 6.00 | 0.00 |

**
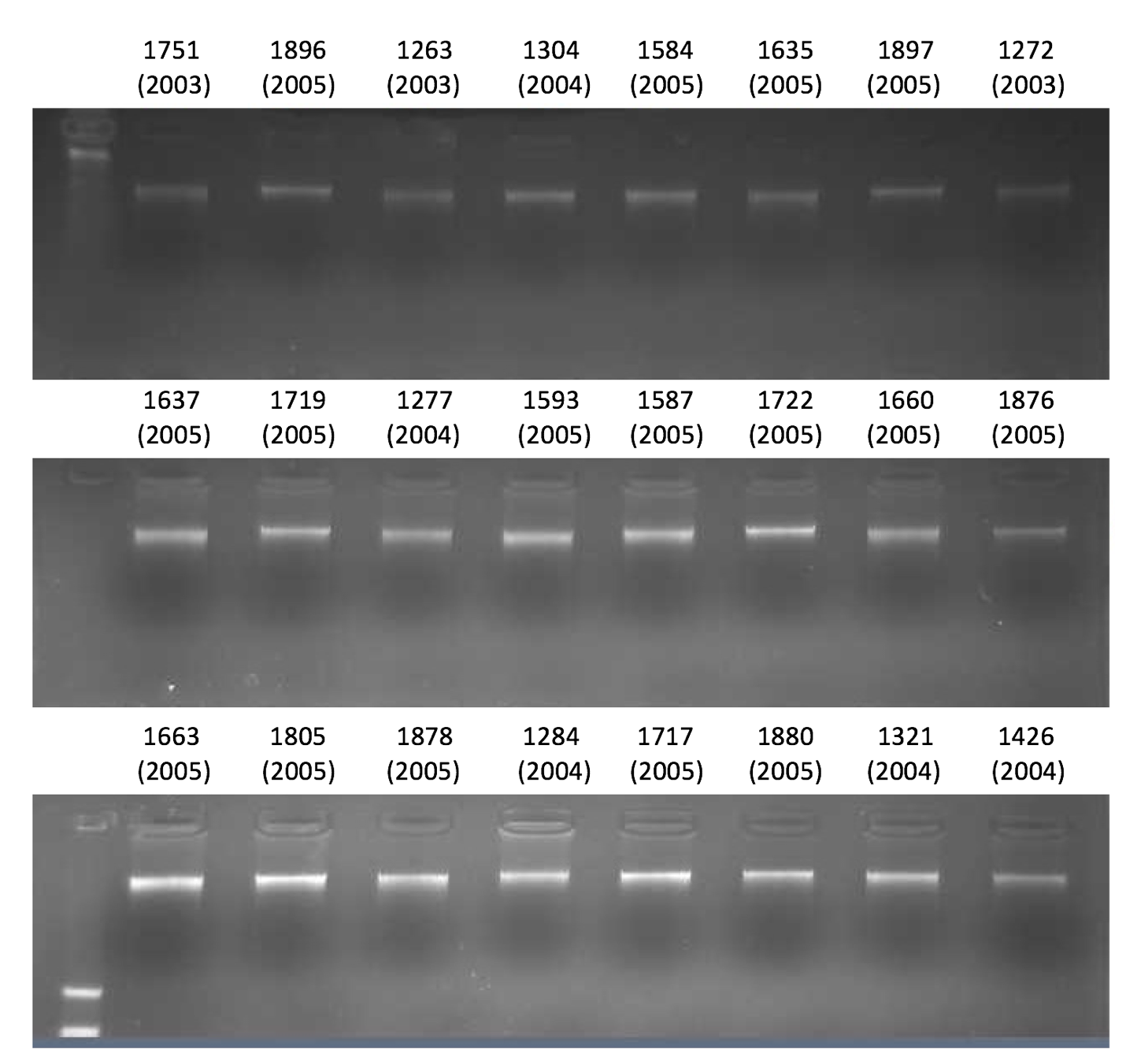
**

**Figure S1** Examples of DNA extractions from older Seychelles warbler samples (sample year in brackets). We found no evidence for differences in DNA integrity with sample age.


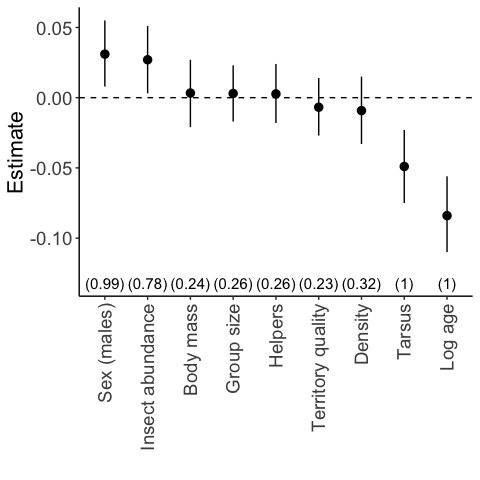


**Figure S2** Model averaged estimates and 95% cofidence intervals for social and ecological environmental variables in relation to RTL in Seychelles warblers.


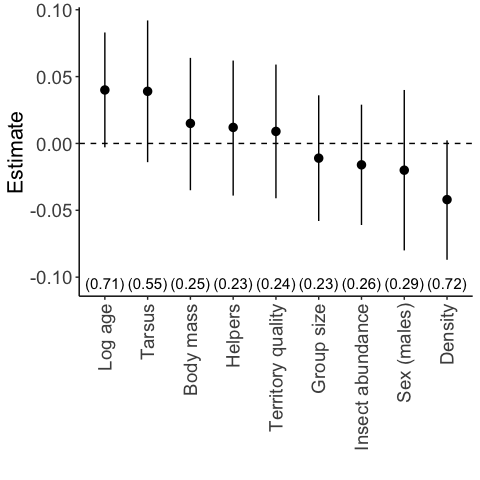


**Figure S3** Model averaged estimates and 95% cofidence intervals for social and ecological environmental variables in relation to $\Delta$RTL in Seychelles warblers, using longitudinal data.
